# Exercise in the treatment of clinical anxiety in general practice – a systematic review and meta-analysis

**DOI:** 10.1186/s12913-018-3313-5

**Published:** 2018-07-16

**Authors:** Elizabeth Aylett, Nicola Small, Peter Bower

**Affiliations:** 1 Thaxted Surgery, Margaret Street, Thaxted, Dunmow, Essex, CM6 2QN England; 2 NIHR School for Primary Care Research, Division of Population Health, Health Services Research and Primary Care, School of Health Sciences, the University of Manchester, Williamson Building, Oxford Road, Manchester, UK

**Keywords:** Anxiety, Panic, Social phobia, Mood, Exercise, Walking, Jogging, Physical activity, Treatment, Randomised controlled trials, Review, Meta-analysis, General practice

## Abstract

**Background:**

Anxiety disorders are common, yet treatment options in general practice are often limited to medication or CBT. There is a lack of evidence for the effectiveness of exercise in the treatment of anxiety in patients who present to general practice and also about the intensity of exercise required to lead to improvement. The aim of this systematic review was to assess the use of exercise versus waiting list control groups in the treatment of anxiety and also to assess the benefit of high intensity exercise vs low intensity exercise. Long term follow up scores were also analysed. We included patients who met diagnostic criteria for anxiety disorders or had clinically raised anxiety levels on a validated rating scale and performed a subgroup analysis of the outcomes between the two groups. The intervention was any aerobic exercise programme carried out for at least two weeks, or exercise carried out at high intensity for at least two weeks. The comparison groups were either a waiting list control group or low intensity exercise.

**Method:**

Systematic review of randomised controlled trials. Three databases were searched; CENTRAL, Medline and Embase. Outcome assessment was based on validated anxiety rating scales. The quality of the studies was appraised according to the Cochrane Risk of Bias tool. Effect sizes were calculated using the standardised mean difference.

**Results:**

Fifteen studies were identified with a total of 675 patients. Nine trials had participants with diagnosed anxiety disorders and six trials had participants with raised anxiety on a validated rating scale. Aerobic exercise was effective in the treatment of raised anxiety compared to waiting list control groups (effect size − 0.41, 95% CI = − 0.70 to − 0.12). High intensity exercise programmes showed greater effects than low intensity programmes. There was no significant difference in outcomes between groups of patients with diagnosed anxiety disorders and patients who had raised anxiety on a rating scale. Conclusions were limited by the small number of studies and wide variation in the delivery of exercise interventions.

**Conclusion:**

Exercise programmes are a viable treatment option for the treatment of anxiety. High intensity exercise regimens were found to be more effective than low intensity regimens. The results have implications for the use of exercise schemes in General Practice.

**Electronic supplementary material:**

The online version of this article (10.1186/s12913-018-3313-5) contains supplementary material, which is available to authorized users.

## Background

Current options for the treatment of anxiety in general practice include psychotherapy such as cognitive behavioural therapy (CBT), or anxiolytic medication such as selective serotonin reuptake inhibitors (SSRIs). Many patients do not want to take anxiolytic medication and would rather turn to lifestyle measures in the first instance as medication brings the possibility of side effects such as nausea. Exercise as a treatment modality has the benefit of being relatively free from side effects while also producing other improvements in health such as weight loss and reduction in blood pressure. It also has the advantage that patients can schedule sessions around their working life rather than needing to take time away from work to attend sessions with a therapist, making it potentially more accessible to patients in addition to being cost-effective to deliver.

Although exercise is a potentially therapeutic option for the treatment of anxiety disorders in general practice, the use of exercise to treat anxiety varies widely and there is little guidance available about the intensity of exercise required to produce a significant improvement [1]. Many GPs have access to “exercise on prescription” schemes where patients receive a subsidised training programme at a local gym. Evidence of the efficacy, and the optimal intensity of the exercise for the treatment of anxiety is necessary in order to inform the structure of exercise schemes that could be offered by GPs.

Previous trials have found exercise to be effective in the treatment of anxiety and both physiological and psychological pathways have been proposed [2]. Physiological mechanisms may include alterations in the serotonergic and noradrenergic pathways; in 1991 Broocks et al. found that 5-hydroxytriptamine turnover is increased in physical activity, while other studies have found that increases in atrial natriuretic peptide are associated with decreased anxiety levels [3–5]. Exposure to the physiological effects of exercise provokes anxious feelings in some individuals and is a reason why many anxiety sufferers are reluctant to undertake exercise (anxiety sensitivity). It has been proposed that exposure to physical training increases tolerance to these symptoms and decreases anxiety sensitivity [6]. Engagement with exercise may lead to an increased sense of self-efficacy as patients see an increase in their ability to cope with the physiological challenges of exercise [7]. Another psychological theory is that of ‘emotion action tendencies’; patients with anxiety disorders tend to withdraw from social situations and engaging in exercise represents a change in social behaviour. Finally, the Distraction Theory posits that exercise may provide “time out” from daily activities and decrease anxious rumination, allowing the patient to think anxiolytic thoughts instead [8].

There have been several previous reviews which addressed the subject of the effectiveness of exercise in the treatment of anxiety in both healthy and clinical subjects. However, not all of the reviews encompass the broad range of patients who present to primary care [9–20]. In addition, very few previous reviews have addressed the question of the optimal intensity of exercise required to effect an improvement in anxiety, which is an important question for patients undertaking a programme of physical activity. Those that did explore this issue were inconclusive [13, 14, 16].

Two recent systematic meta-analyses and one systematic review evaluated the use of aerobic exercise in the treatment of clinical anxiety disorders and limited the participants to those that were formally diagnosed with anxiety disorders according to the Diagnostic and Statistical Manual of Mental Disorders (DSM-IV) [17–19]. In 2012, Jayakodi et al. conducted a systematic review of eight randomised trials of exercise for patients with clinical anxiety disorders [17]. They found an improvement in anxiety symptoms in the groups treated with exercise but concluded that the intensity of exercise needed was unclear. Another meta-analysis in 2013 identified seven trials of exercise in the treatment of diagnosed anxiety disorders [18]. This review found the only trials to report an improvement in anxiety scores were those in which aerobic exercise was compared to a waiting list control group (effect size = − 1.42) rather than alternative treatments such as CBT or medication [18] . Both of these meta-analyses limited the participants to those with formally diagnosed anxiety disorders and the number of studies included was small. A systematic review by Asmundson et al. in 2013 discussed the use of anxiety in diagnosed anxiety disorders and found that the limited evidence available was encouraging regarding the use of exercise as therapy, however their review only included trials of patients with formally diagnosed anxiety disorders and did not include a meta-analysis [19].

The study populations included in other previous reviews are varied, with some reviews including healthy participants, some including participants with raised anxiety scores and others using trials of patients with a wider range of psychiatric illnesses such as depression. Three reviews which used healthy subjects found that exercise was effective in reducing anxiety symptoms among this group of people [9–11]. Two large meta-analyses by Petruzello, in 1991 (one hundred and twenty four studies) [13] and Wipfli in 2008 (forty nine studies) [14], included healthy subjects and those with raised anxiety levels. Results of these reviews gave effect sizes of − 0.48 and − 0.34 respectively for exercise in alleviating anxiety symptoms. A meta-analysis of participants with chronic disease also found exercise to be beneficial in this group of patients [12]. Two further reviews included patients with a range of co-morbid mental health disorders such as depression; these reviews also demonstrated a decrease in anxiety levels after exercise [15, 16].

The study reported here aims to assess the use of exercise in anxiety in order to inform the more widespread use of exercise as a treatment for the patients who typically present in primary care. The review uses a systematic review and meta-analysis to assess the efficacy of exercise programmes for the treatment of patients with anxiety levels higher than the healthy population, including patients who have raised anxiety scores, as demonstrated on a validated rating scale, but not necessarily been diagnosed according to strict diagnostic criteria. Studies where participants had raised anxiety sensitivity have also been included. This is a broader range of participants than in the previous reviews of anxiety disorders, and is representative of the undifferentiated group of anxiety sufferers that present to general practice, where a formal diagnosis has often not been made prior to initial consultation, [21]. The publication of new trials allowed the inclusion of a larger number patients compared to previous reviews of anxiety patients [17–19]. In addition, an analysis of results from studies comparing high intensity exercise to low intensity exercise is included.

## Methods

The protocol for this systematic review was published with the Prospero International Register of Systematic Reviews at http://www.crd.york.ac.uk/PROSPERO (registration number 42014013932).

### Literature search strategy

The search strategy for studies included in this review was restricted to online databases and was conducted using the Simplified Search Strategy detailed by Royle and Waugh [22]. This strategy utilises a search of the Cochrane Central Register of Controlled Trials (CENTRAL), using variants of the word random in all fields. The search is then repeated in MEDLINE and EMBASE. This strategy was shown to have 94% sensitivity in finding trials compared to other methods of searching [22].

There was no time limit on the searches and reference lists of the studies and reviews identified were also searched to detect further studies. The full search strategy that was run in CENTRAL, EMBASE and MEDLINE is given in Additional file 1.

### Study selection

Title and abstract screening was performed independently by two reviewers in April 2015 and September 2015 respectively. Following this initial screening, the full texts of the identified articles were retrieved, and reviewed against the inclusion/exclusion criteria by the first reviewer. A proportion of the full text screening (10%) was performed by a second reviewer independently with excellent inter-rater agreement of 95% (kappa coefficient = 0.99). Any queries regarding inclusion or exclusion of studies were considered by a third author.

Reviews of interest were identified by searching the Cochrane Database of Systematic Reviews as well as running the same search strategy detailed above for reviews as well as trials.

### Inclusion and exclusion criteria

Inclusion criteria were:

1. Randomised controlled trials.
2. Adults (aged > 18 years old) with anxiety levels high enough to meet the accepted threshold for clinically raised anxiety, raised anxiety sensitivity on a validated rating scale, or a formal DSM diagnosis of a specific anxiety disorder.
3. Training regimens of at least two weeks of regular exercise sessions. For inclusion in the high intensity group, the lower threshold of exercise intensity was a minimum of 60% HR max or 60% VO_2_ max.

Exclusion criteria were:

1. Studies where the control groups undertook an alternative active intervention that was not also given to the exercise group. If counselling or medication was used in intervention as well as control groups then trials were included.
2. Trials of patients that were primarily suffering from depression.
3. Trials of patients whose primary medical problem was a chronic medical condition such as cancer or heart disease.
4. Non-English language publications.

### Data extraction

The data extracted from the papers included the following information:

1. Details of the exercise intervention including the type of exercise, length of programme and number of exercise sessions per week. Intensity of exercise was measured as percentage of maximal heart rate (HR max) or percentage of maximal oxygen consumption (V0_2_ max).
2. Types of control groups (waiting list control or low intensity exercise control group)
3. Number of participants in the groups.
4. Diagnostic criteria used in each trial; this was either the DSM criteria for anxiety disorders or the rating scale used to measure anxiety levels.
5. Time spent with the therapist was also extracted for both intervention and control groups in order to give an indication of possible attention bias.
6. The post-intervention anxiety scores in participants.
7. Long term follow up measurements. To be counted as “long term”, the measurement was required to be taken at least two months post-intervention.

The data was extracted by the first author using a data extraction form in Microsoft Excel, and the accuracy of the data extraction from all fifteen studies was verified independently by a second reviewer. One trial was subsequently excluded as the method for calculating the effect size given in the paper was not clearly the same method used for the calculation in the other papers (Merom et al.) [23]. For this reason, the results were excluded from the meta-analysis due to potential inconsistency in the calculations.

### Risk of Bias

Data were extracted using Cochrane Collaboration’s Review Manager 5.3 which also provides a template for the evaluation of bias [24]. The items used in this template include an evaluation of whether the paper discusses the following methodological issues; random sequence generation and allocation concealment, blinding of participants and personnel, blinding of outcome assessment, incomplete data and selective reporting. In addition an evaluation was made on each study of the amount of time spent by participants in each group with the therapist. The assessment of bias was made by one author at the study selection stage. The overall quality of evidence was evaluated using the GRADE approach which gives an overall assessment of the quality of evidence for outcomes in systematic reviews, based on the risk of bias, inconsistency in results, indirectness, imprecision and publication bias [25].

### Analysis

For continuous data, effect sizes were calculated as the Standardised Mean Difference based on post-test scores between intervention and control groups. These effect sizes were then weighted prior to aggregation using an inverse variance and fixed effects model and combined to give an overall effect size using the statistical software programme StatsDirect [26]. We examined outcomes from studies comparing exercise versus waiting list control, and from studies comparing high and low intensity exercise. We also compared the outcomes from studies which included patients with an anxiety disorder according to diagnostic criteria with those which included patients with raised anxiety levels on rating scales (but no formal diagnosis).

## Results

**Fig. 1:**
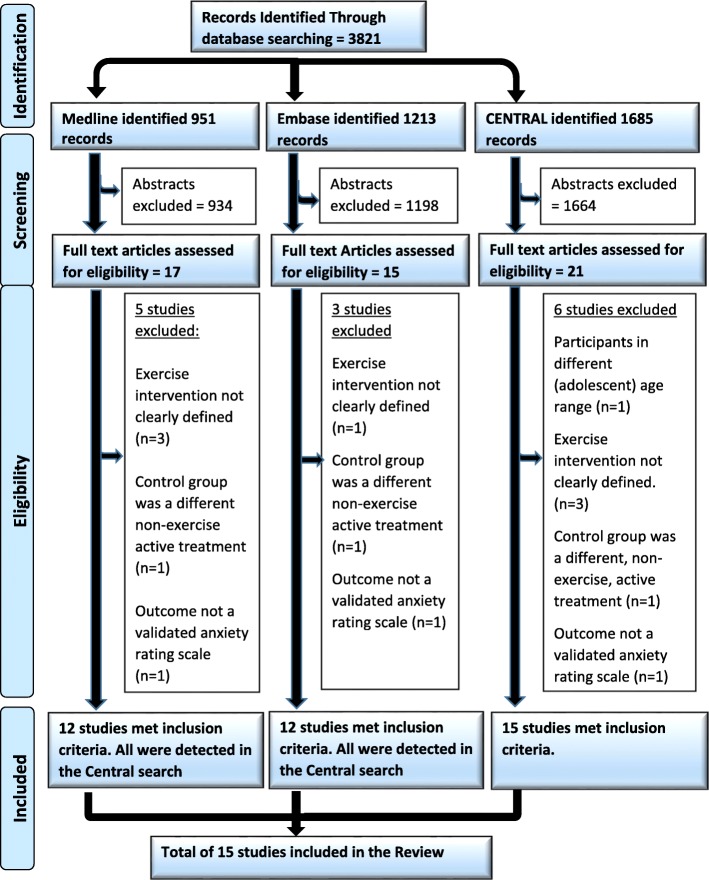
PRISMA flow chart outlining the process of study selection

Figure 1 presents the PRISMA flow chart for study selection. Screening of abstracts left twenty one studies for review of the full text. After analysis of these articles, seven studies were excluded because they did not meet the criteria for inclusion [23, 27–32]. A table of these excluded studies can be found in Additional file 2.

### Characteristics of studies comparing exercise vs waiting list control groups

There were ten randomised controlled trials that compared exercise groups with non-exercise control groups with a total of 422 patients (Table 1).

**Table 1:** Characteristics of Studies; Exercise Group vs Waiting list Control Group

Author	Study Size	Diagnostic criteria	Intervention	Control Group	Were groups matched for time spent with the trainer?	Long Term Follow up	Outcome measure
*Herring 2012 (a)*[35]	*n* = 20	Patients meeting DSM IV criteria for Generalised Anxiety Disorder.	Supervised aerobic exercise, twice per week for 8 weeks.	Untreated waiting list group..	No	No	Penn State Worry Questionnaire (PSWQ)
*Herring 2012(b)*	*n* = 20	Patients meeting DSM IV criteria for Generalised Anxiety Disorder	Resistance Exercise.	Untreated Waiting list control group	No	No	Penn State Worry Questionnaire (PSWQ)
*Jazaieri* et al.*. 2012*[34]	*n* = 54	Patients with DSM IV social anxiety disorder as measured on the ADIS-IV-L scale	3 sessions of aerobic exercise per week over two months. 2 individual and 1 group. Intensity and Heart rate not measured. Duration 10 weeks	Untreated SAD group	No	No	Liebowitz Social anxiety self-report scale (LSAS-SR), Social Interaction Anxiety Scale (SIAS-S)
*Smits* et al.*. 2008* [36]	*n* = 35	Elevated Anxiety Sensitivity > 25 on the Anxiety Sensitivity Index	Six 20 min aerobic exercise sessions over two weeks on a treadmill.	Untreated waiting list control group.	No	Follow up at 3 weeks	Anxiety Sensitivity Index (ASI) Beck Anxiety Inventory (BAI)
*Merom* et al.*. 2007* [23]	*n* = 74	Patients meeting DSM IV criteria for Generalised Anxiety Disorder, Panic Disorder or Social Phobia	8 week programme of 30 min walking sessions, measured with pedometer, increasing to 5 sessions per week. Intensity not measured. Also had CBT and education	CBT and education	Yes, sessions with the exercise trainer were matched with CBT educational sessions in control group.		The Depression Anxiety Stress Scale (DASS 21)
*Mailey* et al.*.........., 2010* [37]	*n* = 51	Students with clinically raised anxiety levels	Internet delivered programme, over a 10 week time period. Activity measured with an accelerometer. Counselling.	Counselling	No	No	State trait anxiety Inventory (STAI trait)
*Broman-Fulks, 2008* [33]	*n* = 24	Score higher than non-clinical mean on Anxiety sensitivity score	Aerobic exercise 6 twenty min. Sessions over a 2 week time period. Subjects fitted with polaris monitor. HR 60–90% maximum	waiting list control	No, controls attended to fill in the ASR-I only.	No	Anxiety Sensitivy Index (ASI)
*Wedekind 2010* [38]	*n* = 37	DSM IV criteria for panic disorder with or without agoraphobia.	Exercise for 45 mins 3 times per week, initially walking increasing to running. Duration was 10 weeks.	Relaxation plus placebo	No	No	Clnical Global Impression Scale (CGI)
*Brooks* et al *1998* [39]	*n* = 31	Patients meeting the DSM III criteria for panic disorder and Agoraphobia	A four mile route to be walked, progressing to running 3 times per week for 10 weeks. Also one training session per week.	Placebo group	No	No	HADS, Panic and Agorsphobia scale (P&A)
*Villaverde* et al *2012* [40]	*n* = 36	Menopausal women with clinically raised anxiety according to the HRSA scale	Six month programme of 6o min sessions 3 times per weeks including aerobic and resistance exercise performed to 60–80% HR max.	Waiting list control group	No	No	Hospital Anxiety and Depression Scale (HADS)
*Medina* et al *2015* [41]	*n* = 60	Elevated Anxiety Sensitivity > 25 on the Anxiety Sensitivity Index	Six 20-min moderate intensity aerobic exercise sessions over two weeks (three sessions per week),	Untreated waiting list control group.	No	No	Anxiety Sensitivity Index (ASI) Beck Anxiety Inventory (BAI)

The numbers of participants ranged from twenty four in the smallest [33] to seventy four in the largest study [23]. Five trials were included in the meta-analysis [33–37] and five were eligible but the data presentation did not allow them to be included in the meta-analysis [23, 38–41]. Of the ten studies, five trials included patients with formally diagnosed anxiety disorders [23, 34, 35, 38, 39] and five included patients with clinically raised anxiety, or anxiety sensitivity, on a recognised rating scale [33, 36, 37, 40, 41].

Exercise interventions included running, walking, treadmill training and supervised aerobic training sessions. Intensity of the exercise was not recorded in all of these trials as some of the programmes included exercise undertaken outside of the lab. In these cases the exercise was recorded with pedometers and accelerometers worn by the participants. In those trials were intensity was recorded, the high intensity level ranged from 60 to 90% of HR max.

The duration of the programmes were as follows; four trials were ten weeks long [34, 37–39], two were of eight weeks duration [23, 35], three programmes were of two weeks duration [33, 36, 41] and one lasted for six months [40]. Frequency of exercise sessions varied from five times per week in one study [23], four times a week one study [39], three times per week in six studies [33, 34, 36, 38, 40, 41], twice a week in one study [35] and one study did not have a defined number of sessions [37].

There was a non-active waiting list control group in six studies [33–36, 40, 41], two studies used psychological therapy in both exercise and control groups [23, 37], and two studies used a non-active control group with placebo pills [38, 39]. The intervention and control groups were only matched for time spent with a therapist in one of the trials [23].

A range of outcome measures were used in the trials and are given in Table 1. The broad range of rating scales used to measure anxiety outcomes in the trials reflect the spectrum of anxiety disorders represented in the review including conditions such as panic disorder, General Anxiety Disorder, raised anxiety sensitivity and also more generally raised anxiety levels.

### Characteristics of studies of high intensity vs low intensity exercise

There were five studies with a combined number of 253 participants which compared high intensity exercise to low intensity exercise [42–46], (Table 2).

**Table 2:** Characteristics of Studies comparing High Intensity to Low Intensity Exercise

Author	Study Size	Diagnostic criteria	Intervention	Control group	Were groups matched for time spent with the trainer?	Long term follow up	Outcome measure
*Broman-Fulks, 2004* [43]	*n* = 54	Students with a score of 25 or more (0.75 SD over the mean) on the Anxiety Sensitivity Index	Six 20 min treadmill sessions at high intensity for 2 weeks. Polaris heart monitor to assess HR. 60–90% HR max.	Six 20 min walking at low intensity over two weeks.	Yes	Measurements repeated one week later	Anxiety Sensitivity Index (ASI), and State Trait Anxiety Inventory (STAI trait)
*Sexton* et al *1989* [44]	*n* = 40	Non-psychotic Inpatients with anxiety disorders	Jogging, 30 mins 3 or 4 times per week for 8 weeks at 70% HR max	Walking for 3 or 4 times per week over 8 weeks at a comfortable speed.	Yes	Yes, 6 months later	State Trait Anxiety Inventory (STAI trait)
*Steptoe* et al *1989* [45]	*n* = 33	Volunteers with anxiety levels in the “borderline” or “definite” range on HADS scale and/or raised score on the Profile of Moods States.	10 weeks of one supervise and three unsupervised sessions. 20 mins of continuous walking at 60–65% HR max.	10 weeks of one supervise and three unsupervised sessions. Strength, mobility and flexibility not above 50% HR max.	Yes	Yes at 3 months	State Trait Anxiety Inventory (STAI trait)
*Gaudlitz* et al.*. 2015* [46]	*n* = 47	Participants had Panic Disorder according to DSMIV criteria	30 mins on treadmill 3 times per week for 8 weeks to 70% VO2 max.	Low intensity stretching exercises for 30 mins 3 times per week for 8 weeks	Yes	Yes at 7 months	Hamilton Anxiety Scale (Ham-A), BAI
*Martinsen* et al*, 1989* [42]	*n* = 79	Patients meeting DSM III criteria for panic disorder with or without agoraphobia, Generalised anxiety disorder or social phobia.	Brisk walking or jogging to 70% VO2 max, Trained 3 times per week for 8 weeks.	Anaerobic training - strength, flexibility and relaxation - low intensity.	Yes	No	Comprehensive Psychopathological Rating Scale (CPRS), Phobic Avoidance Rating Scale (PARS)

One study identified by the search was eligible for the review but could not be included in the meta-analysis due to the data presentation [42]. Three studies included patients with formally diagnosed anxiety disorders [42, 44, 46] and two studies included patients with general anxiety scores above the clinical mean [43, 45].

High intensity intervention groups undertook aerobic exercise such as jogging, treadmill exercise or walking which was performed to a minimum of 60% HR max or 70% VO_2_ max. Low intensity control groups undertook less strenuous aerobic exercise such as comfortable walking or stretching and flexibility exercises.

The longest programme was of ten weeks duration [45], three programmes were eight weeks long [42, 44, 46] and one trial was just two weeks long [43]. All of the studies comparing high and low intensity exercise were matched for time spent with the therapist. No trials utilised psychological therapies such as CBT in intervention or control groups.

### Risk of Bias in studies

**Fig. 2:**
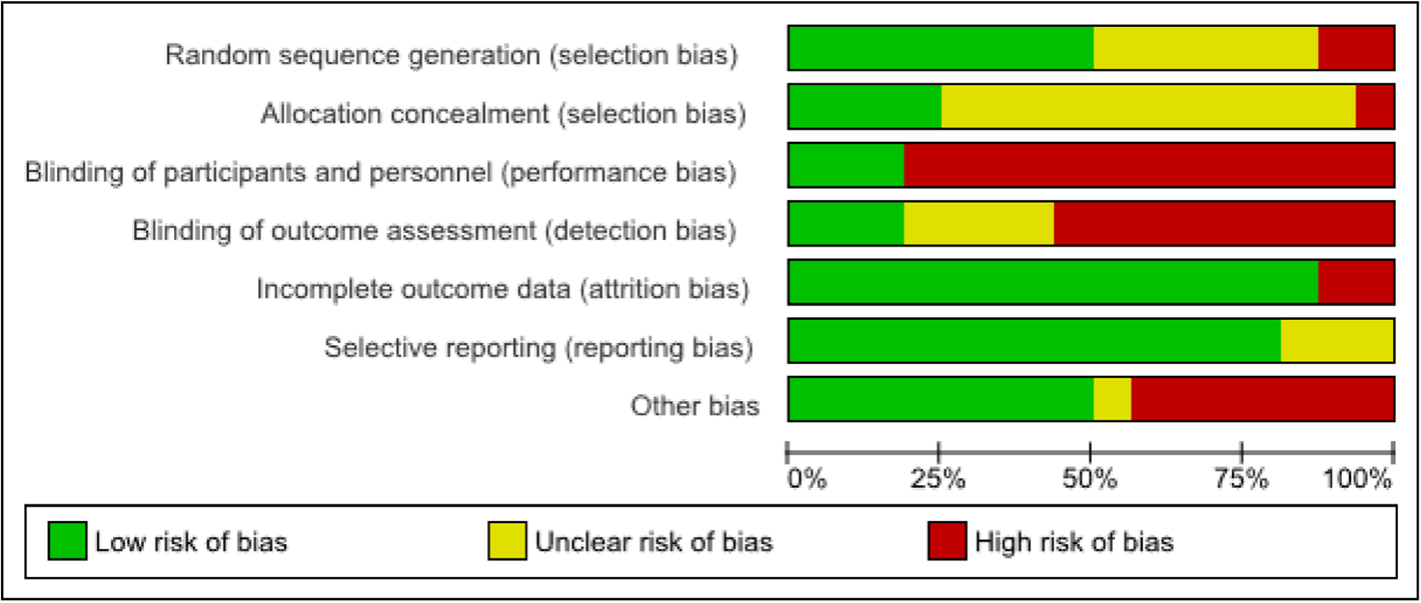
Risk of bias summary: Overall risks of bias items for included studies

The overall risk of bias for all the different studies included in the review is demonstrated in Fig. 2.

The main source of bias in the studies was that of the lack of blinding of participants and personnel. This was true of the majority of trials and is a common problem where participants engage in an active intervention. Only three studies addressed this problem; in the study by Gaudlitz et al. [46], participants in exercise groups of differing intensity were blinded as to the group they were in and so were the personnel assessing outcome. This study was judged overall to be the best quality for minimising the risk of bias in all categories. Control participants in the study by *Brookes* et al were given placebo medication and outcome assessors were also blinded [39]. Participants in the trial by Broman-Fulks et al were unaware of the intervention used in the other group [33]. Attention bias, where the groups were not matched for the time spent with a supervisor, was also a difficulty in some trials. In these instances, the improvement could potentially be due to the time spent with the trainer, rather than the exercise itself. Of the fifteen studies, only eight had matched the time spent with a therapist between the groups [23, 38, 39, 42–46]. Evaluation of risk of bias in each trial is given in Table 3.

**Table 3:** Risk of Bias in each trial

Study	Random Sequence	Allocation Concealment	Blinding Participants	Blinding Outcome	Incomplete Outcome	Selective Reporting	Attention Bias
Herring et al., 2012 [35]	Blocked randomisation stratified according to medication use.	Clinicians performing the initial assessment blinded to allocation	Participants not blinded X	Not blinded. X	All allocated subjects completed the study.	All outcomes were reported.	Low intensity and high intensity groups matched for therapist time but not waiting list control group.
Jazaieri et al., 2012 [34]	Randomised using Efrons randomisation procedure	No information (?)	Participants not blinded (X)	No information regarding assessor blinding (?)	No difference in attrition between groups.	Patients self-reported the quantity of exercise performed. (?)	patients were not matched for time spent with an instructor. (X)
Smits et al. 2008 [36]	Computer Generated Random Sequence	No allocation concealment (X)	Participants were not blinded (X)	. Not blinded. (X)	No difference in attrition between groups.	All outcomes were reported.	Attention bias, patients were not matched for the spent with an instructor. (X)
Merom et al. 2007 [23]	Computer Generated Randomisation	Performed by independent research centre	Participants not blinded (X)	Not Blinded (X)	No difference in attrition between groups.	All outcomes were reported.	Groups had matched time with therapist.
Mailey et al., 2010 [37]	No information about method of randomisation (?)	No information (?)	Participants not blinded (X)	Not blinded (?)	Very little attrition in both groups.	All outcomes were reported.	Intervention and control groups not matched for time (X)
Broman-Fulks, 2008 [33]	No information about the method of randomisation (?)	No information (?)	Participants were blinded as to the group they were in	Not Blinded (X)	All allocated subjects completed the study.	All outcomes were reported.	Attention bias, patients were not matched for the spent with an instructor. (X)
Wedekind 2010 [38]	Blocked randomisation	Allocation performed by the hospital pharmacist	Participants not blinded (X)	Blind rater used to eliminate expectation differences (?)	No significant difference in drop outs between groups.	All outcomes were reported.	Subjects in the control group received time with a therapist doing relaxation training.
Brooks et al. 1998 [39]	No information about method of randomisation (?)	No information (?)	Participants were blinded.	Investigators were blinded	Similar attrition rate	All outcomes were reported.	Time matched with therapist across groups
Villaverde et al. 2012 [40]	Details given regarding the randomisation procedure	No information (?)	Participants not blinded (X)	Not Blinded (X)	Similar attrition rate between groups.	All outcomes were reported.	No time matching between groups (X)
Medina et al. 2015 [41]	Not mentioned in the method section. (X)	No information (?)	Participants not blinded (X)	Not Blinded (X)	Similar attrition rate	All outcomes were reported.	No time matching between groups (X)
Broman-Fulks, 2004 [43]	No information about method of randomisation (X)	No information (?)	Participants not blinded (X)	Not Blinded (X)	No attrition in the groups	All outcomes were reported.	Matched for time
Sexton et al. 1989 [44]	No information about the method of randomisation (?)	No information (?)	Participants not blinded (X)	Not Blinded (X)	higher no. of dropout in the high intensity group (X)	All outcomes were reported.	Less risk of attention bias as both active interventions.
Steptoe et al. 1989 [45]	No information about the method of randomisation (?)	No information (?)	Participants not blinded (X)	Not Blinded (X)	Similar attrition rate between groups.	Some outcomes were not reported (?)	Participants in both groups had similar time with therapists
Gaudlitz et al. 2015 [46]	Randomised using block allocation	Assessment staff were blinded	Participants were blinded as to nature of the study	Study staff were blinded	Low attrition rate and all subjects accounted for	All outcomes were reported.	Time matched with therapist across groups
Martinsen et al., 1989 [42]	Randomised using block allocation	No information (?)	Participants not blinded (X)	Assessors were not blinded	Low attrition rate	All outcomes were reported.	Groups were time matched

The quality of evidence was moderate for all outcomes and is demonstrated in Table 4. The GRADE score was downgraded by one in all outcomes due to the lack of blinding of participants in most of the trials and also for the possibility of attention bias due to time spent with the supervisor being therapeutic in itself.

**Table 4:** Summary of Findings Table for Grade outcomes

Aerobic Exercise compared to Placebo for the treatment of Anxiety				
Patient or population: Patients with raised anxiety levels on a validated rating scale or diagnosed with Anxiety disorders. Intervention: Aerobic exercise for anxiety, Comparison: Non exercise Control Groups.				
Outcomes	Risk with Aerobic exercise for anxiety	№ of participants (studies)	Quality of the evidence (GRADE)	Comments
Improvement in anxiety scores in patients who exercised compared to no exercise. Assessed with: Evidence based anxiety rating scales follow up: range 2 weeks to 10 weeks	0.41 SMD lower (0.70 lower to 0.12 lower)*	194 (6 RCTs)	⨁⨁⨁◯MODERATE ^a,^	The risk of bias noted is due to the lack of blinding of participants to the intervention in most of the studies. Also bias due to time spent with supervisor
High intensity exercise compared to low intensity exercise for Anxiety Disorders				
Patient or population: Anxiety Disorders Intervention: High intensity exercise Comparison: low intensity exercise				
Outcomes	Risk with High intensity exercise	№ of participants (studies)	Quality of the evidence (GRADE)	Comments
Results of groups who undertook high intensity exercise compared with those who undertook low intensity exercise. Assessed with: Evidence based anxiety rating scales follow up: range 2 weeks to 10 weeks	0.38 lower (0.68 lower to 0.08 lower)*	174 (4 RCTs)	⨁⨁⨁◯MODERATE ^a^	The risk of bias noted is due to the lack of blinding of participants to the intervention in most of the studies. Time with supervisor was matched in these trials
Long term High intensity exercise compared to Long term low intensity exercise for Anxiety Disorders				
Patient or population: Anxiety Disorders, Intervention: Long term High intensity exercise,Comparison: Long term low intensity exercise				
Outcomes	Risk with Long term High intensity exercise	№ of participants (studies)	Quality of the evidence (GRADE)	Comments
Improvement in anxiety levels in high intensity groups compared to low intensity groups over a longer time period. Assessed with: Evidence based anxiety scores follow up: range 3 months to 7 months	- 0.33 SMD lower (0.74 lower to 0.08 lower)*	96 (3 RCTs)	⨁⨁⨁◯MODERATE ^a^	The risk of bias noted is due to the lack of blinding of participants to the intervention in most of the studies. Time with supervisor was matched in these trials

^a^GRADE Working Group grades of evidence High quality: We are very confident that the true effect lies close to that of the estimate of the effect, Moderate quality: We are moderately confident in the effect estimate: The true effect is likely to be close to the estimate of the effect, but there is a possibility that it is substantially different, Low quality: Our confidence in the effect estimate is limited: The true effect may be substantially different from the estimate of the effect, Very low quality: We have very little confidence in the effect estimate: The true effect is likely to be substantially different from the estimate of effect

*The risk in the intervention group (and its 95% confidence interval) is based on the assumed risk in the comparison group and the relative effect of the intervention (and its 95% CI)

*CI* Confidence interval; *SMD* Standardised mean difference

### Analysis

#### Exercise vs waiting list control group

**Fig. 3:**
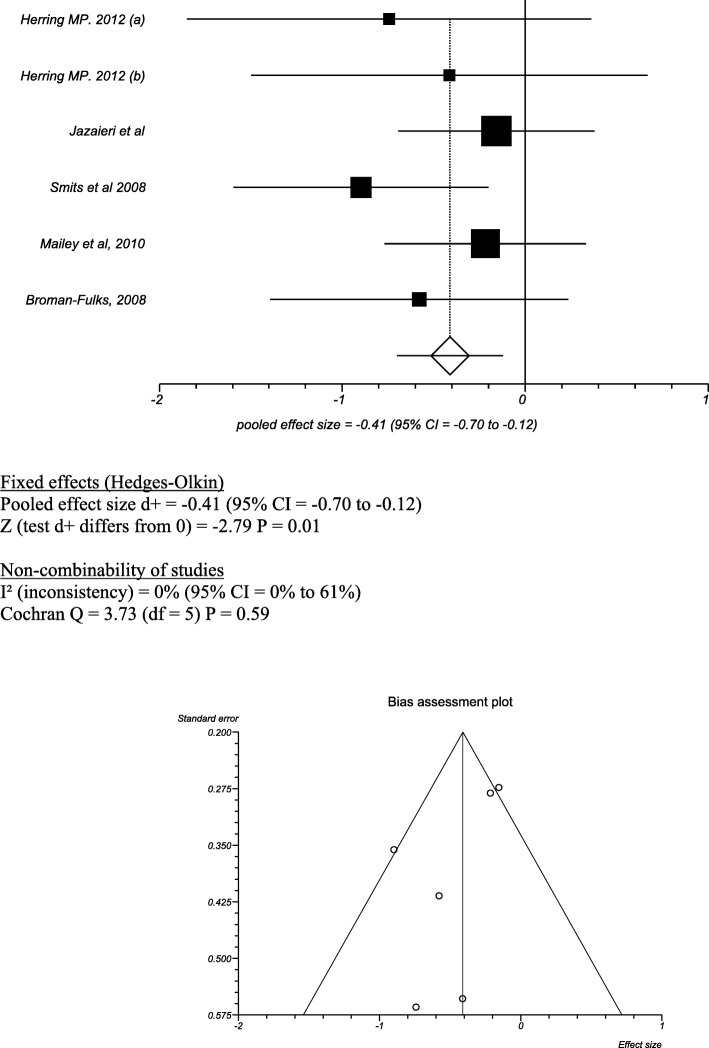
Exercise vs waiting list control group

The results from the meta-analysis of Exercise group vs Waiting list control group gave an effect size of − 0.41 (95% CI = − 0.70 to − 0.12), where a negative effect size denotes an improvement in anxiety scores. The heterogeneity given by the I^2^ test was 0% (95% CI = 0 to 61%)) (Fig. 3).

Of the studies that were excluded from the numerical meta-analysis due to data presentation, three of the trials also found that exercise was significantly more effective than placebo in reducing anxiety symptoms [38–40]. One study found that exercise did not lead to a statistically significant improvement in symptoms compared to patients in a relaxation group [37]. Table 5 shows the results for the Exercise vs Waiting List Control Group.

**Table 5:** Results for Exercise vs Waiting List Control Group

	Mean (SD)	Total	Mean (SD)	Total	SMD (95% CI)	Outcome
*Broman-Fulks, 2008* [33]	27.92 (15.36)	12	41 (25.68)	12	− 0.60 (− 1.42, 0.22)	Exercise lead to significant reductions in exercise sensitivity compared to the untreated group
*Jazaieri, 2012* [34]	61.41 (28.64)	25	65.42 (21.37)	29	−0.16 (− 0.69, 0.38)	Exercise lead to non-significant reductions in anxiety compared to the untreated group.
*Herring, 2012(a)* [35]	59.3 (7.38)	10	65.5 (7.62)	5	−0.79 (− 1.71, 0.13)	Exercise lead to non-significant reductions in anxiety compared to the untreated group,
*Herring 2012(b)* [35]	61.10 (10.01)	10	65.5 (7.62)	5	−0.44(−1.53,0.65)	Resistance exercise lead to non-significant reductions in anxiety compared to the untreated group,
*Smits, 2008* [36]	10.19 (6.54)	16	18.26 (10.24)	19	−0.92(−1.62,-0.22)	Exercise and exercise + CBT both lead to statistically significant reductions in both the ASI and BAI
*Merom, 2008* [23]	Not Reported	38	Not Reported	36	−0.16 (− 0.77, 0.45)	CBT and exercise lead to a greater, non-significant improvement in DASS-21 scores compared to CBT and education.
*Mailey, 2010* [37]	44.05 (18.02)	26	47.23 (9.29)	25	−0.22(− 0.77,0.33)	There was a small and non-significant improvement in anxiety and depression in the exercise group.
*Wedekind 2010* [38]	Not Reported	20	Not Reported	17	Not Reported	Exercise and relaxation both lead to reductions in anxiety, not statistically significant. F value = 3.7
*Brooks* et al *1998* [39]	11.5	16	22.8	15	Not Reported	Exericise lead to significant improvement in symptoms but not as effective as Clomipramine F value = 13.4
*Villaverde* et al.*.. 2012* [40]	16.76	17	15.02	19	Not Reported	There was a small non-significant improvement in the exercise group.
*Medina* et al *2015* [41]	Not Reported	Not Reported	Not Reported	Not Reported	Not Reported	There was greater but non-significant improvement in anxiety sensitivity for those in Exercise compared to Waiting List control F value = 26.7

### High intensity vs low intensity exercise

**Fig. 4:**
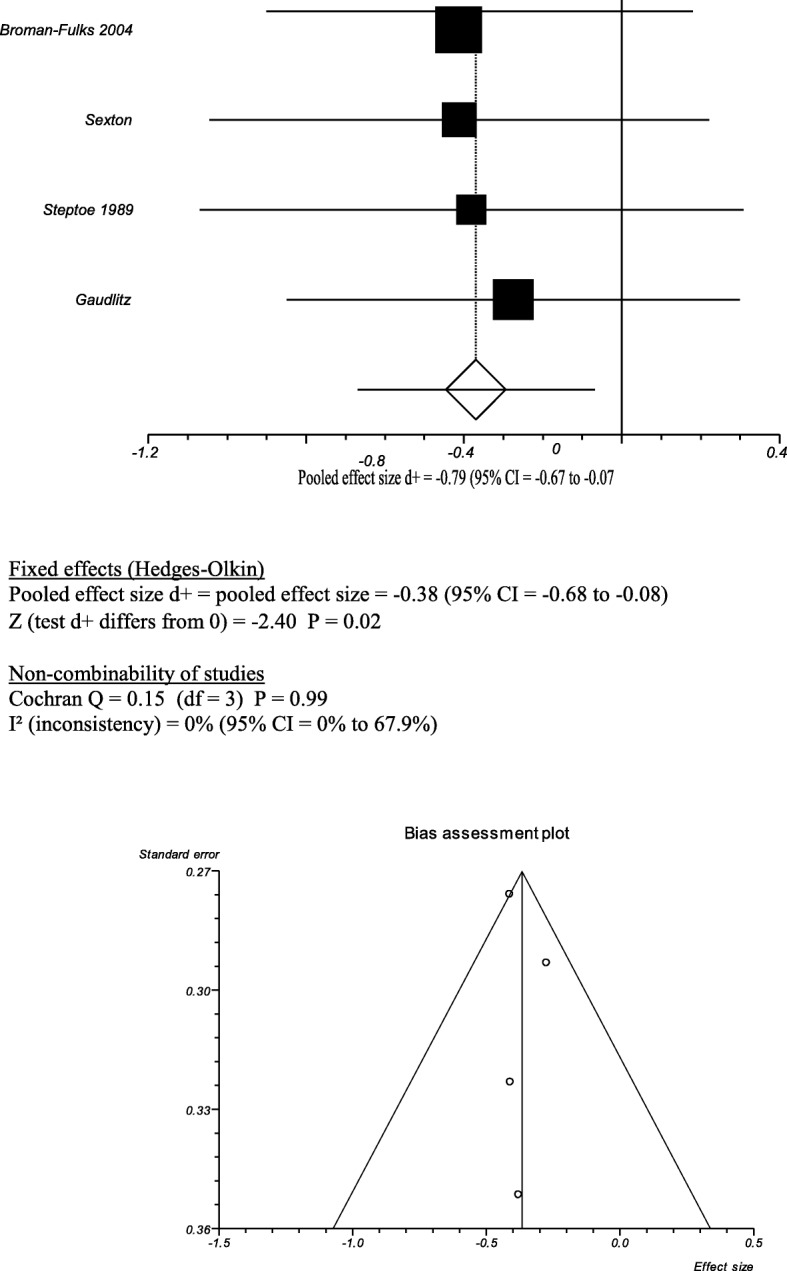
High Intensity vs Low Intensity Exercise

Figure 4 shows the meta-analysis of the results of four trials which each included a comparison of high intensity exercise with low intensity exercise. The pooled estimate of differences found in those four studies gave an effect size of − 0.38 (95% CI = − 0.68 to − 0.08), suggesting that high intensity exercise training is more effective at lowering anxiety levels than lower intensity training (Fig. 4). The heterogeneity given by the I^2^ test was 0% (95% CI (0 to 67.9%).

Drop-out rates were greater in the high intensity group in the study by Sexton and equal in the studies by Broman-Fulks and Steptoe et al*.* [43–45]. In the study by Gaudlitz et al. there more non-completers in the low intensity groups than in the high intensity group [46]. Of the trials that were excluded from the meta-analysis, Martinsen et al found that there was no difference in anxiety reduction between walkers and joggers, but that there was a higher drop-out rate in the jogging group (*P* > 0.1) [42]. Table 6 shows the results for the High Intensity Exercise vs Low Intensity Exercise Groups.

**Table 6:** Results for high intensity exercise vs low intensity exercise

	Mean (SD)	Total	Mean (SD)	Total	Std Mean Difference	Outcome
*Broman-Fulks,2004* [43]	25.03 (9.71)	29	28.56 (6.01)	25	−0.42(−0.96,0.12)	High intensity exercise led to more rapid reductions in anxiety sensitivity than low intensity exercise
*Sexton, 1989* [44]	41.2 (11.3)	17	46.2 (12.0)	23	−0.42(−1.05,0.22)	Both jogging and walking led to a reduction in anxiety. Jogging led to a greater reduction than walking but this was not statistically significant.
*Steptoe, 1989* [45]	42.3 (11.5)	17	46.5 (9.1)	16	−0.39 [−1.08, 0.30]	The moderate exercise led to greater reductions in anxiety than the low intensity attention placebo group.
*Gaudlitz, 2015* [46]	11.9 (7.1)	24	14.3 (9.4)	23	−0.29 [− 0.86, 0.29]	Higher Intensity Exercise and Low Intensity exercise both led to a reduction in anxiety scores. There was further improvement of anxiety over time with a medium-sized effect in the endurance training group, but not in the control group.
*Martinsen* et al *1989* [42]		36		43		At the end of the study both groups had achieved significant reductions in scores compared with admission values The differences between groups were small and not statistically significant *P* > 0.1

### Results from long term follow up scores

Three studies from the high intensity vs low intensity group gave measurements for long term follow up scores [44–46]. They all found that the reduction in anxiety from exercise was maintained several months after the training in both high intensity and low intensity groups. Two studies found that high intensity exercise lead to bigger reductions in the long term [45, 46] and one study reported a similar reduction between high intensity and low intensity groups [44]. The combined effect size from these three studies was − 0.30 (95% CI = − 0.72 to 0.12) (Table 7). The heterogeneity given by the I^2^ test was 18.2% (95% CI = 0 to 77.5%).

**Table 7:** Results of long term follow up scores

	Length of follow up	High Intensity exercise Mean (SD)	Low Intensity exercise Mean (SD)	St Mean difference
Sexton, 1989 [44]	6 months	43 (10.1)	42 (16.8)	0.07 (−0.55,0.70)
Steptoe, 1989 [45]	3 months	39.9 (10.5)	46.6 (10.8)	−0.60 (−1.56,0.36)
Gaudlitz, 2015 [46]	7 months	8.5 (7.3)	14.2 (9.8)	−0.66 (−1.31,0.00)
				−0.30 (− 0.72,0.12)

### Subgroup analysis of results from patients with raised anxiety levels and those with diagnosed anxiety disorders

**Fig. 5:**
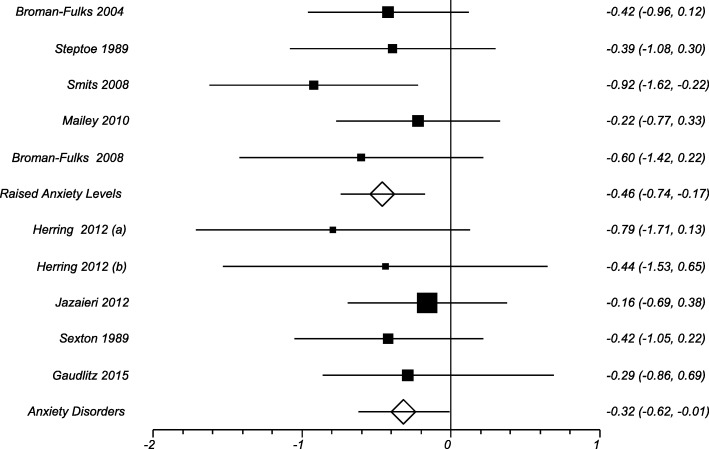
Results from Studies of patients with Raised Anxiety Levels and Studies of Patients with Anxiety Disorders

There were seven trials identified where patients had raised anxiety levels based on a validated rating scale [33, 36, 37, 40, 41, 43, 45], five of these trials gave data which could be included in the meta-analysis [33, 36, 37, 43, 45]. The overall effect size in this group of participants was − 0.46 (− 0.74 to − 0.17) (Fig. 5). Both of the trials not included in the meta-analysis also showed a reduction in anxiety scores in the exercise group compared with the control group [39, 40]. There were eight trials identified were participants had formally diagnosed anxiety disorders [23, 34, 35, 38, 39, 42, 44, 46], of which four were included in the meta-analysis [34, 35, 44, 46]. The overall effect size in this group of patients was a smaller reduction in anxiety symptoms of − 0.32 (0.62 to − 0.01) There was no significant difference in the mean reduction of symptoms between the two groups of trials *P* = 0.24 (− 0.39 to 0.19).

## Discussion

### Principal findings

This review identified ten randomised controlled trials which assessed the use of Exercise versus Waiting list control groups [23, 33–41] and five randomised controlled trials comparing High Intensity to Low Intensity exercise for the treatment of anxiety [42–46]. Participants in these trials had a diagnosis of an anxiety disorder according to DSM criteria [23, 34, 35, 38, 39, 42, 44, 46] or had high anxiety levels / anxiety sensitivity levels on a validated anxiety rating scale [33, 36, 37, 40, 41, 43, 45]. Results showed that exercise is more effective than waiting list control group with a moderate effect size of − 0.41 (95% CI = − 0.70 to − 0.12) which was statistically significant. High Intensity exercise was found to be more effective than low intensity exercise with a significant effect size of − 0.38 (95% CI -0.68 to − 0.08). Follow up scores in high intensity exercise indicate that improvement in anxiety levels is maintained for several months after training with a non-significant effect size of − 0.33 (− 0.74, 0.08). It was disappointing that so few studies had long term follow up scores but although the effect is formally not significant, the magnitude is similar.

Continued engagement with the exercise programme is a potential problem with higher intensity exercise regimens. One study found that there were higher drop-out rates with the groups that undertook high intensity compared to low intensity training [44]. This study stated that the participants found the programmes too strenuous. This clearly has implications for the structure of the exercise programme as it is necessary to maximise the number of participants who complete the regimen. Given the finding that high intensity regimens are more effective than low intensity regimens, exercise programmes need to be carefully tailored to the individual, especially patients with high anxiety sensitivity levels, in order to maximise the benefit from exercise while minimising the risk of the patient dropping out.

### Subgroup analysis

The pooled effect size from five trials which included patients with formally diagnosed anxiety disorders was − 0.32 (− 0.62 to − 0.01) which was not significantly different from the effect size in patient with raised anxiety levels which was − 0.46 (95% CI -0.74 to − 0.17). *P* = 0.24 (− 0.39 to 0.19). This finding needs replication with a larger number of studies. Any differences in outcome between these groups could potentially be attributed to worse baseline scores in patients with anxiety disorders, however differences in rating scales used in these studies makes baseline severity difficult to compare. This could be relevant when managing different groups of patients as the more heterogeneous group of patients with raised anxiety levels may be more representative of the patients who present in primary care compared to those attending hospital out-patients clinics with formally diagnosed anxiety disorders. However, we would reiterate that caution should be exercised in the interpretation of the subgroup analysis due to the small number of studies and the fact that other differences between the studies could account for the results.

### Comparison with other reviews

Previous reviews of clinical anxiety disorders have given conflicting results about the efficacy of exercise. A recent review by Bartley et al*.* [18] concluded that exercise is not effective in the treatment of anxiety disorders but this review used active control groups for comparison; for example, the trial by Jazieri et al [34] had both a mindfulness control group and also an untreated waiting list control group; Bartley et al. used the mindfulness based stress reduction control group as a comparison, whereas the current study uses the untreated control group; as mindfulness is an active treatment then the effect size from this comparison might reasonably expected to be smaller. In the sub-analysis of studies comparing Exercise groups to untreated waiting list control groups, Bartley et al*.* actually found a large effect size of − 1.42 (0.8, − 2.04). This is a larger effect size than in this study (− 0.41), however only two studies were included in the sub-analysis in that particular review compared to seven results in the current study [36, 39]. Jayakody et al identified eight trials and found that exercise is effective as an adjunct treatment for anxiety [17]. All eight trials in that review were identified by the search in the current study but two were excluded due to study design, one at the screening stage [47] and one during assessment for eligibility [28]. The effect size found in this review was similar to that found in the current meta-analysis and also the review by Petruzello et al, where the effect size was − 0.34 [13] and Long et al who found an effect size of − 0.36 [10].

The current, updated, meta-analysis adds to existing evidence that exercise is an effective treatment for anxiety, particularly the patient group that are likely to present in general practice who may present with a range of anxiety problems. The inclusion of patients without a formal DSM diagnosed anxiety disorder allowed the results of an additional three trials to be included compared to other recent reviews of clinical patients and the subgroup analysis suggests that exercise may be particularly helpful in this group of patients. Results from this review also confirm the findings of previous studies of healthy subjects which found that higher intensity exercise is more effective at relieving anxiety symptoms than low intensity exercise [11, 12, 14, 15].

### Strengths and weaknesses

The difficulty in drawing conclusions from this meta-analysis is that there is still a small number of randomised trials on clinical patients in this subject area. Five studies were excluded from the meta-analysis due to the data presentation, which, given the small number of studies in the review could make a difference to the overall results, although the results from the excluded trials were in general agreement with those included. There were four studies comparing aerobic exercise to waiting list control which were not included in the meta-analysis [38–41]; these trials all found that exercise led to an improvement in anxiety symptoms compared to the untreated control group and the results were statistically significant in three of them. There was only one study excluded from the meta-analysis of results from trials comparing High Intensity versus Low Intensity exercise; the trial by Martinsen et al. [42] compared joggers with walkers and found the reduction in symptoms was almost identical in both high intensity and low intensity groups. This result is in contrast with that of the four studies included in the meta-analysis which found that higher intensity exercise was more effective than lower intensity exercise [43–46].

The large variability in the type of exercise undertaken in the intervention and control groups makes comparison between trials difficult, especially when comparing high intensity to low intensity exercise. In the exercise vs waiting list control group, the exercise intervention was similar in most of the studies (jogging or treadmill exercise). Lack of time matching with a supervisor could be a source of bias in these trials as the social encouragement from a supervisor could be therapeutic in itself. In the exercise vs waiting list control group, only one trial matched the time spent with a supervisor in the control group. In the high intensity vs low intensity exercise group, all the trials matched the supervision times between intervention and control groups. The review by Bartley et al. found that time matched studies had lower effect sizes than those which were not time matched [19].

Lack of blinding of participants to the interventions used was an issue in the majority of these studies. Only three trials made attempts to blind the subjects as to the interventions used in the different groups [33, 39, 46] and two trials used assessors which were blinded [39, 46]. If patients are aware of the group to which they are assigned this could lead to reporting bias. While generally recognised as being important to minimise bias, blinding of therapist- rated outcomes may be less important in trials where the outcomes are reliably measured by subjects using evidence based self-rating questionnaires, which was the case in most of these studies.

A limitation of this review is that we were unable to involve two independent reviewers across the full study selection process. The use of two investigators may reduce the possibility of rejecting relevant reports where the selection or rejection of an article requires difficult judgments. In this review, the study selection process was continued by one reviewer after establishing an excellent inter-rater agreement between two reviewers across a significant proportion (10%) of the studies identified by the searches. For the data extraction, two reviewers checked the data from all fifteen studies which were identified in the review.

A limitation in the review methodology was the potential for bias in study selection from the exclusion of non-English language papers and also from the risk of publication bias.

The exclusion of non-English language papers could, in theory, lead to a reporting bias. However, inclusion of non-English language papers in the Medline search only yielded a further 27 articles. Of these, only one study was relevant; this was a Hungarian review, which found a small to moderate effect for the use of exercise in the prevention and treatment of anxiety. There were no further randomised trials identified [48]. A further limitation in study selection is the risk of Publication Bias, where there is a chance that only trials with positive results will be published, this can invalidate the results of a meta-analysis and is a particular risk in reviews where the number of studies is small [49] We sought to limit publication bias by searching grey literature using the TRIP database, but this did not yield any further suitable randomised trials for inclusion in the review.

Statistical analysis of the results in this study was based on the method recommended in the Cochrane Handbook [50] and utilized in the software program Revman 5.3 [22], comparing post-test scores from intervention and control groups in each trial. Such methods assume comparability of baseline scores on outcome measures. All participants were randomised, so any differences in baseline scores within trials would be limited, although baseline imbalance is possible, especially when the number of patients included is small. An alternative method of analysis is to use gain scores where the change in anxiety score in each study is pooled together to get the overall effect size. However, there is no clear evidence to suggest that one method has any advantage over the other, and the use of gain scores may require additional assumptions when data on change are not presented (https://effectivehealthcare.ahrq.gov/sites/default/files/pdf/choice-of-mean_white-paper.pdf)

### Implications for general practice

The principal findings from this updated review confirm those from previous studies which found that exercise is a useful and realistic option for treatment of raised anxiety levels in General Practice. Given that anxiety is such a widespread condition, the availability of a treatment option which is both relatively free of side-effects and also has the potential to be continued by patients is a welcome addition to the options available to General Practitioners and their patients. However, the implementation of exercise needs to be evaluated carefully. The TREAD study, in which patients were offered training as a treatment for depression found that the strategy offered was not effective, although in this trial participants in the both intervention and control groups were referred to exercise schemes [51]. Given that high intensity exercise was found to be more effective in the current review, the level of supervision required to implement exercise to the optimal intensity needs further study.

The utility of widespread exercise on prescription schemes on the NHS depends on the cost-effectiveness of such programmes. An economic evaluation was carried out in the Wales National Exercise Referral Scheme comparing the cost of a lifestyle intervention which included exercise with improvements in quality of life. The authors calculated that the cost effectiveness ratio was £12,111 per QALY, falling to £9741 if patients contributed £2 per session. This easily meets the threshold of £30,000 per QALY used as a benchmark by NICE in determining affordability of treatments [30].

Future studies should focus on the questions that need to be answered in order to translate the finding that exercise is effective for anxiety into the optimum design and delivery of exercise on prescription schemes. Although this study provides evidence that higher intensity exercise is more effective than lower intensity exercise, this needs further clarification; more well designed clinical trials are needed where the intensity and type of exercise is clearly defined in both intervention and control groups. In addition more research is needed on the benefit of exercise in different levels of anxiety disorders as some studies have shown that this varies according to the specific diagnosis. For example Merom found that improvement was greatest in patients with social phobia [23]. The optimum length of exercise schemes also needs further evaluation and economic assessment. In addition, the benefit of exercise as a stand-alone therapy could be further evaluated against treatment with psychotherapy and pharmacotherapy in primary care patients with raised anxiety levels [39].

## Conclusion

This study adds to existing evidence that aerobic exercise is effective in the treatment of patients with clinically raised anxiety such as those seen in primary care. Higher intensity exercise may have an advantage over lower intensity exercise in bringing about an improvement in anxiety scores but the conclusions are limited in view of the small number of studies and varying exercise regimens that were tested. The increased efficacy of higher intensity regimens should be considered when tailoring exercise programmes to individual patients. Patients with raised anxiety levels benefit as much from exercise than those who have received a formal anxiety diagnosis. These findings confirm that exercise represents an effective treatment and should be more available for referral from General Practice. Future trials could address the use of exercise in specific anxiety disorders and evaluate the optimum intensity of exercise to promote completion of the exercise programme.

## Additional files

Additional file 1: Full search strategy. (DOCX 12 kb)

Additional file 2: Table of Exclusions. (DOCX 12 kb)
