# Supplementary material for: Exercise in the treatment of clinical anxiety in general practice – a systematic review and meta-analysis
Source: BMC Health Serv Res. 2018 Jul 16;18:559. doi: 10.1186/s12913-018-3313-5 (PMC6048763; doi:10.1186/s12913-018-3313-5)
Supplement: Supplementary file 1 — Full search strategy. (DOCX 12 kb) [file 12913_2018_3313_MOESM1_ESM.docx]

**Additional File 1; Full Search Strategy**

This is the strategy that was run in Medline, Embase and CENTRAL.

1. Anxiety ti,ab
2. Panic ti,ab
3. Social phobia ti,ab
4. Agoraphobia ti, ab
5. Mood ti,ab
6. Mental health ti,ab
7. 1-6 /OR ti,ab
8. Exercise ti,ab
9. Walking ti,ab
10. Jogging ti,ab
11. Physical activity ti,ab
12. OR / 8-11 ti,ab
13. Treatment
14. Therapy
15. OR /13-14
16. Random* af
17. 7 AND 12 AND 15 AND 16
