# Supplementary material for: Exercise in the treatment of clinical anxiety in general practice – a systematic review and meta-analysis
Source: BMC Health Serv Res. 2018 Jul 16;18:559. doi: 10.1186/s12913-018-3313-5 (PMC6048763; doi:10.1186/s12913-018-3313-5)
Supplement: Supplementary file 2 — Table of Exclusions. (DOCX 12 kb) [file 12913_2018_3313_MOESM2_ESM.docx]

**Additional File 2; Table of Exclusions**

| **Paper** | **Reason for Exclusion** |
| --- | --- |
| Knapen *et al* [28] | Intervention was not clear as to the intensity of the aerobic exercise component in each exercise programme. Patient group was “non-psychotic inpatients” rather than anxiety patients. |
| Hovland *et al* [30] | Control group was a different, non-exercise, active treatment |
| Oeland *et al* [31] | Outcome not a validated anxiety rating scale |
| Murphy *et al* [29] | Exercise Intervention not clearly defined |
| Lambert *et al* [27] | The intervention was not clearly defined as an exercise intervention. Exercise was part of a broader lifestyle intervention |
| Brown *et al*  [26] | Participants in adolescent age range |
| Merom *et al* [33] | Method of effect size calculation unknown and not enough data given in the paper to allow our own calculation. |
